# Mercury content in the Siberian tiger (*Panthera tigris altaica* Temminck, 1844) from the coastal and inland areas of the Russia

**DOI:** 10.1038/s41598-021-86411-y

**Published:** 2021-03-25

**Authors:** N. Ya. Poddubnaya, G. P. Salkina, L. S. Eltsova, E. S. Ivanova, A. Yu. Oleynikov, D. D. Pavlov, V. Kh. Kryukov, O. Yu. Rumyantseva

**Affiliations:** 1 Cherepovets State University, Cherepovets, Russia; 2 FSBI United Directorate Lazovsky State Nature Reserve them L. G. Kaplanova, and the National Park “Call of the Tiger”, Lazo, Russia 692980; 3 Institute for Water and Environmental Problems SB RAS, Khabarovsk, Russia; 4 Institute for Biology of Inland Waters RAS, Yaroslavl, Russia

**Keywords:** Ecology, Zoology, Ecology, Environmental sciences

## Abstract

Being a global pollutant, mercury can originate from both natural as well as anthropogenic sources. Coastal marine atmospheric fog is considered a potential source of ocean-derived monomethylmercury (MMHg) to coastal terrestrial ecosystems. However, the ratio between mercury appearing through natural processes and that from the results of human activity is unclear. We assumed that the total mercury content in the fur of tigers would differ depending on the distance from the sea. Here we show that the average mercury content in tigers from the coast (0.435 ± 0.062 mg kg^−1^) is significantly different from tigers from the inland area (0.239 ± 0.075 mg kg^−1^), (p = 0.02). We found that the content of mercury in the fur of tigers is largely dependent of natural processes rather than human activity. We assume that the levels of mercury in coastal ecosystems in the south of the Russian Far East reflect the position of the region relative to the deep faults of the East Pacific Platform. Obtained data indicate that environmental risks associated with mercury pollution currently exist, but do not pose a serious threat to Siberian tigers.

## Introduction

Key studies of Weiss-Penzias et al. ^1–3^ have shown that terrestrial ecosystems are likely to receive ocean-derived monomethylmercury (MMHg) through the coastal marine atmospheric fog. This seems incredible and yet very important, as the problem of mercury pollution of inanimate and living matter on the planet has been a hot spot in science and politics for more than half a century. It does not lose its severity primarily because it is only being solved locally, hindered by financial constraints on the global scale. At the same time, there are usually no pollution surveys and therefore no data on the mercury (Hg) content in living objects even in sites where intensive mining of gold or mercury-containing minerals was carried out in the last 150–200 years (see ^4,5^ for example). Approximately half of the total global anthropogenic mercury emissions occur in Asian countries ^6^.

The Russian Far East is a region where gold was mined or is still being mined at different locations at different times, or where there are deposits of mercury-containing minerals ^7^. In the south of the Russian Far East, these are mainly the Khanka–Khasan–Grodekovsky massif and the Fadeevsky ore-placer cluster ^7–10^. Apparently, it is historically associated with deposits located in China.

At the same time, the southern regions of the Russian Far East are unique in the number of Mesozoic relics—species that require special conservation measures. The list of rare animals contains one of the largest felids on the planet—the Siberian tiger (*Panthera tigris altaica* Temminck, 1844). Tigers are the apex predators of ecological pyramids, and therefore have the potential to accumulate mercury through bioaccumulation.

The Siberian subspecies of the tiger preys on 85 species of animals, with five species of ungulates constituting the main part of the diet: red deer (*Cervus elaphus* Linnaeus, 1758), sika deer (*C. nippon* Temminck, 1838), roe deer (*Capreolus pygargus*) and musk deer (*Moschus moschiferus* (Linnaeus, 1758)), wild boar (*Sus scrofa* Linnaeus, 1758), much less often—bears (*Ursus arctos arctos* Linnaeus, 1758; *U. thibetanus* G. Cuvier, 1823), pheasant (*Phasianus colchicus* Linnaeus, 1758), hares *Lepus timidus* (Linnaeus, 1758), *L. mandshuricus* Radde, 1861), badger (*Meles leucurus* (Hodgson, 1847)) and other small animals ^11–16^. Similar to jaguars ^17,18^ the Amur tigers can eat reptiles, fish and small mammals (e.g. chipmunks, voles, mice), sometimes they can prey on domestic animals, such as dogs, pigs and cows ^13^. When live food is scarce, tigers feed on carrion; its share in the tiger’s diet can reach 8.9% ^15^. However, they do it reluctantly ^13^, like other large felids (for example, see ^19^.

We examined the mercury content of the Siberian tiger—the uppermost link of the food network of coastal and inland ecosystems in the south of the Russian Far East.

## Results and discussion

This is the first study to evaluate the mercury content in the fur of Siberian tigers in the Far East of Russia. It is a commonly recognized fact that fish are the main source of mercury entering the organism of predators and the trophic network of the ecosystem. In some areas, the seasonal abundance of salmonids can provide tigers with protein: masu (*Oncorhynchus masou*) from April to early July, chum (*O. keta*) in late autumn and up to December and pink salmon (*O. gorbuscha*) in July–early October. However, our observations during 1976–2018 (^15^ (Poddubnaya, unpublished data)) and data on mercury content show that tigers do not eat salmon often. Tigers do not hunt the redfin dace (*Tribolodon hakonensis*) a cyprinid fish, moving up in huge swarms from April to June.

Although tigers were never observed to consume fish in mass quantities, as is the case with bears, one would expect that the total concentration of mercury (THg) in the body of tigers from two sections of the Sikhote-Alin (basin drainage of the Sea of Japan and the catchment of the Amur River) would vary depending on the availability of anadromous fish. The rivers of the Sea of Japan basin are shorter and shallower than Amur’s tributaries. Therefore, the likelihood of a tiger catching fish here seems to be higher. However, Amur’s tributaries are richer in fish and the probability of catching fish has to be no less than that observed in the coast. Thus, it can be assumed that the proportion of fish in tiger’s diet on the coast and in the inland region should be similar. Apparently, the consumption of salmon by tigers can be neglected in this analysis.

The minor role of fish in the Siberian tiger diet is further evidenced by comparing its average mercury content with other large felids known to consume fish and other aquatic animals. Thus, individuals of Florida panther (*Puma concolor coryi*), consuming aquatic and fish-eating animals have elevated levels of MMHg—1.62 ± 1.87 mg/kg or 1.84 mg/kg THg ^20^. The average mercury concentration in the jaguar (*Panthera onca)*, mainly preying on fish and alligators, reaches even higher values of up to 4.27 mg/kg (from 2.13 to 7.26 mg/kg) ^21^. The average THg content in the Siberian tiger is 0.383 ± 0.062 mg/kg indicating that it eats little fish, if any.

In the south of the Russian Far East, some ungulates caught by the tiger on the eastern macro slope of Sikhote-Alin periodically go to the sea to lick salt and eat algae. This can lead to some increase in the level of mercury in their tissues and in the tiger along the trophic chain. In addition, ungulates, especially deer, can eat various lichens, including Usnea in the temperate forests.

**Figure 1:**
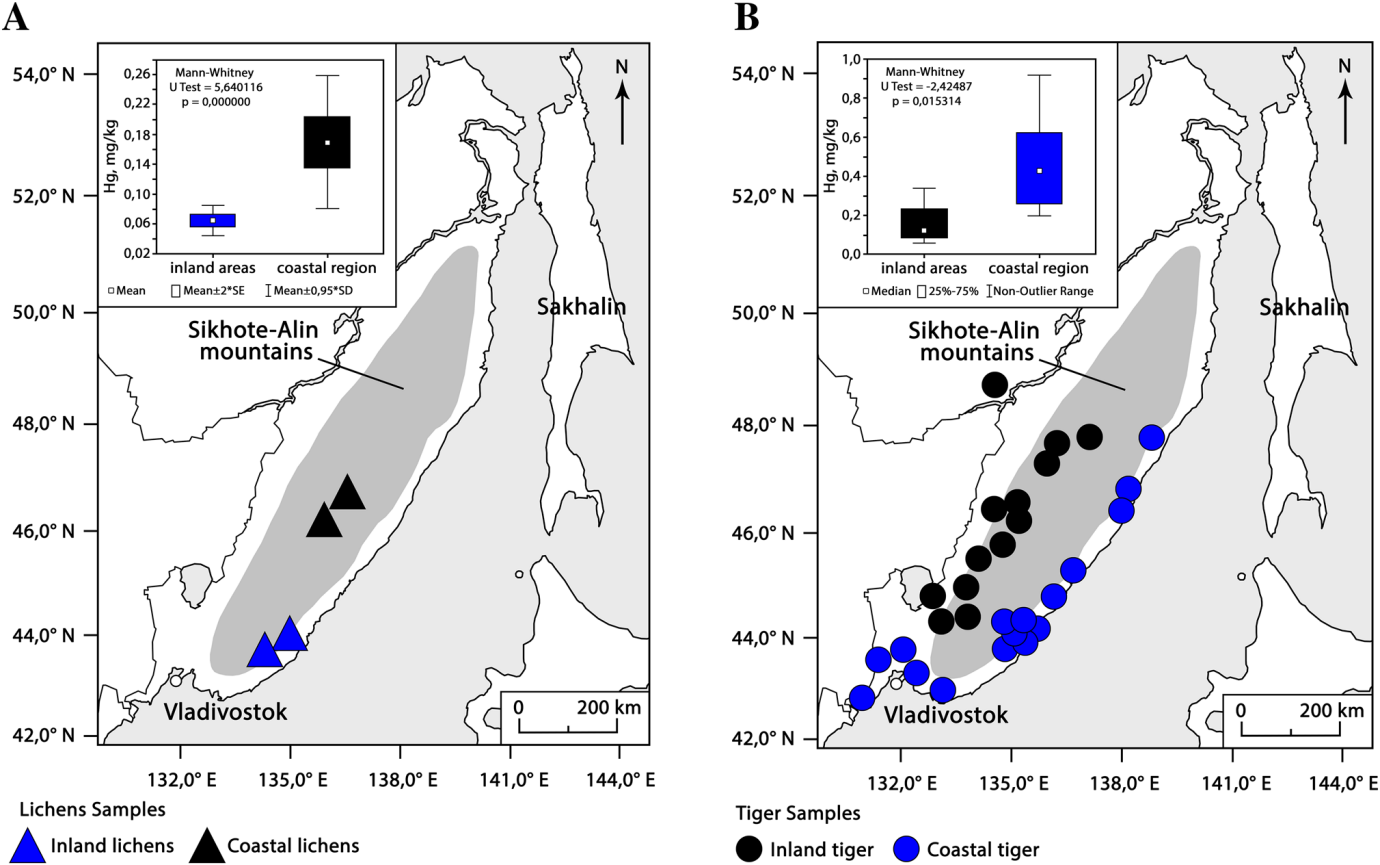
Map of sampling sites and mean values of (**A**) THg concentrations in lichen (*Usnea* sp.) (site names correspond to data in Table 1), (**B**) THg in tiger fur. The blue triangles and circles represent the samples from coastal sub-region and the black—from inland sub-region. Lichen sampling was done in 2019 and tiger fur sampling was done in 2004–2014. The map was generated in Adobe Photoshop CS6, based on a map from the public domain on the site https://yandex.ru/legal/maps_termsofuse/?lang=en.

As we found out, THg in lichens from the coast, where sea fog is observed, was 0.170 ± 0.017 mg kg^−1^ (n = 30), which is 2.6 times higher than the average value for inland areas (0.065 ± 0.004 mg kg^−1^ (n = 24) (Fig. 1A). The absolute values of THg in *Usnea* lichens from the coast in the south of the Russian Far East turned out to be higher than in the *Ramalina menziesii* lichens (from the same order Lecanorales and the same ecological form as *Usnea*) from the coast in California ^3^ (0.138 ± 0.012 mg kg^−1^). It is possible that such differences are related to species-specific features of their thalli. Different species of lichens from the same locality can accumulate different amounts of toxic substances in their thallus. Thus, *Usnea* contained 0.170 ± 0.017 mg kg^−1^, and the mesomorphic evernia (*Evernia mesomorpha*) collected on the same site—0.292 mg kg^−1^(n = 2).

Weiss-Penzias et al. ^3^ do not give the average THg for inland lichens, but they show that the high MMHg content in lichens on the coast is obtained through coastal marine atmospheric fog. We compared our data with those for THg on Bathurst Island ^22^, where the spatial pattern in THg enrichment was very similar to that of MMHg, with enrichment highest at coastal sites and decreasing within 10 km, suggesting similar origins of atmospheric THg and MMHg to lichens. Potential sources of inorganic Hg and MMHg to lichens are diverse (e.g. ^3,22^). MMHg from THg can range from 4.4 to 23% ^3^, therefore special future studies are needed to understand the dynamics of Hg species in lichen.

The average individual THg concentrations in tiger fur samples from the coast ranged from 0.115 to 0.918 mg kg^−1^ (n = 12), on average 0.434 ± 0.067 (Fig. 1B, Table 1), while tiger fur samples from the inland regions (n = 12) had lower concentrations of THg (range from 0.057 to 0.950 mg kg^−1^, average 0.239 ± 0.075); the differences between the means of the two sites were statistically significant (p = 0.01) (Fig. 1B, Table 1).

**Table 1:** Statistics on the subsets of THg concentration data used in this paper.

Dataset	N	Mean	Median	Minimum	Maximum	Lower quartile	Upper quartile	Standard dev	Standard error	Mann–Whitney U test
Coastal male and female tiger	13	0.435	0.424	0.115	0.918	0.255	0.561	0.224	0.062	*U = 2.50 p = 0.01
Inland male and female tiger	12	0.239	0.135	0.057	0.950	0.080	0.285	0.259	0.075	
Female tiger	14	0.331	0.223	0.057	0.918	0.115	0.503	0.264	0.070	U = 0.27 p = 0.78
Male tiger	11	0.354	0.339	0.067	0.950	0.150	0.442	0.259	0.078	
Coastal female tiger	7	0.478	0.481	0.115	0.918	0.214	0.689	0.276	0.104	U = − **2**.**11** p = **0**.**03**
Inland female tiger	7	0.183	0.119	0.057	0.503	0.080	0.232	0.153	0.058	
Coastal male tiger	6	0.385	0.396	0.196	0.622	0.255	0.442	0.151	0.062	U = 1.28 p = 0.20
Inland male tiger	5	0.317	0.150	0.067	0.950	0.081	0.339	0.370	0.165	
Juv tiger	13	0.219	0.170	0.056	0.622	0.110	0.232	0.176	0.049	U = − **1**.**99** p = **0**.**04**
Ad tiger	14	0.422	0.396	0.067	0.950	0.182	0.561	0.282	0.075	
Inland juv tiger	7	0.165	0.110	0.056	0.503	0.057	0.232	0.160	0.061	U = − 1.00 p = 0.32
Inland ad tiger	6	0.295	0.166	0.067	0.950	0.081	0.339	0.335	0.137	
Coastal juv tiger	6	0.281	0.205	0.115	0.622	0.170	0.369	0.187	0.077	U = − **2**.**07** p = **0**.**04**
Coastal ad tiger	8	0.517	0.462	0.255	0.918	0.396	0.625	0.207	0.073	
Coastal juv tiger	6	0.281	0.205	0.115	0.622	0.170	0.369	0.187	0.077	U = − 1.57 p = 0.12
Inland juv tiger	7	0.165	0.110	0.056	0.503	0.057	0.232	0.160	0.061	
Coastal ad tiger	8	0.517	0.462	0.255	0.918	0.396	0.625	0.207	0.073	U = − 1.94 p = 0.05
Inland ad tiger	6	0.295	0.166	0.067	0.950	0.081	0.339	0.335	0.137	
Coastal region lichen	30	0.170	0.152	0.024	0.541	0.121	0.171	0.094	0.017	U = 5.64 p = 0.00
Inland areas lichen	24	0.065	0.059	0.043	0.146	0.053	0.071	0.022	0.004	

All concentrations are in mg kg^−1^.

*Except for four samples with unknown gender.

Statistically significant differences are given in bold.

The average concentrations of THg in the tiger fur of two subregions were lower (0.434 ± 0.067 and 0.239 ± 0.075 mg kg^−1^) than similar values for pumas from California ^3^. In contrast to California, where the average THg content in pumas from the coastal area was three times higher than in animals from the inland areas, in the Russian Far East the average THg content in tiger fur sampled in the area influenced by the sea fog was two times higher than that in comparable samples from inland areas. Although, it is the inland area (the Amur River basin) that is subject to high levels of human activity, including the mining of coal and gold in the past and present, and we could expect higher levels of mercury in living components of ecosystems. The average concentrations of THg in the tiger fur were only from 0.056 to 0.232 mg kg^−1^ (n = 4) near coal and gold mining sites.

Different age classes were sampled in both the coastal and inland areas, and THg concentrations increased with age (adult > young) in both areas (Table 1). This pattern is typical for predatory animals in general and for pumas, in particular ^3^, which is natural due to the cumulative effect and increasing mercury content with age ^22–25^. Differences in the average individual mercury content in individual fur samples of young and adult tigers were significant (p = 0.04) (Table 1). Moreover, the differences between the average mercury content in young and adult tigers were insignificant in the inland site (p = 0.32), while being significant on the coast (p = 0.04) (Table 1).

We did not observe any significant differences in THg concentrations between the sexes (p = 0.86) (Table 1) and between males from the coast and the inland (p = 0.25) (Table 1) as was noted earlier for puma ^3,21^. On the contrary, the average values of mercury in the fur of individuals from the coast were 3.1 times higher than from the inland sites within the group of females, this difference was statistically significant (p = 0.03) (Table 1). These data contradict to what was observed in puma by Weiss-Penzias et al. ^3^. Such feature of female tiger is apparently associated with their shorter migration routes and smaller individual territories compared to males ^26,27^. Unlike males, which can cross the main Sikhote-Alin ridge, females are usually located either on the territory in the zone of sea fog influence, or in the inland areas.

In addition, young females often remain within the territory of their mothers during dispersal ^15^. Rather similar information was obtained for the wild European cat ^28^, where THg in females was about 1.4 times higher than in males, although the differences were not statistically significant. There were no significant differences in THg content between young tigers in coastal and inland areas, as well as in the samples of animals, which died in autumn–winter and spring-early summer (Table 1).

Apparently, preying on land animals does not lead to the accumulation of high Hg levels in felids. The average Hg levels in the fur of a near-water species such as the ocelot (*Felis pardalis*) mainly preying on terrestrial animals, varied in the same range as the tiger: 0.5–1.25 mg kg^−1^ ^29^. Tigers are mainly consumers of the second level and therefore the average content of mercury in their body (0.383 ± 0.062) (Table 1) is lower than in the fur of consumers of the third level such as the pine marten 1.80 ± 1.34 mg kg^−1^ ^30^ or Daubenton’s bat 1.15 ± 0.27 mg kg^−1^ ^31^.

The only sample with a maximum mercury content of 1.402 mg kg^−1^ (age and gender unknown) was from the southwestern Primorye, which is located on the coast and where there are cinnabar deposits nearby. This sample was not used in the total analysis. Interestingly, the available sample of a young female Far Eastern leopard fur (*Panthera pardus orientalis*) from the same site had practically the same mercury content (1.456 mg kg^−1^). Local increased mercury content in the body of tigers can be associated with deposits of mercury-containing minerals. These data do not confuse our understanding of the sources of mercury in the ecosystem; they serve as a signal for a more profound study of natural processes.

Our data on a higher mercury level (THg) in lichens and tigers of the sea coast compared to inland areas may be related to the effects of coastal sea atmospheric fog, a potential source of monomethylmercury (MMHg) produced in the ocean ^3^.

The levels of mercury we found in Siberian tigers from the Russian Far East are about four times lower than the mercury content in the fur and vibrissa of puma from California ^3^. It seems that such differences are related to the position of these regions relative to the zones of deep faults of the mantle formation of the East Pacific platform ^32^. The maximum concentrations of Hg in the near-surface atmosphere are confined to such zones, and the concentrations decrease at a distance from them. California is located closer to such zones, while the south of the Russian Far East is further away.

And the fact that different levels of mercury in ecosystems depend on the distance relative to the deep faults of the East Pacific platform is confirmed, for example, by pink salmon: fish from the Sea of Japan contain much less mercury than fish from the Kuril region closer to the fault zone (from 0.045 to 0.087 mg kg^−1^ wet weight ^31^. At the same time, we must not forget that California is the most populated and one of the most industrially developed states in the USA. However, it seems that natural processes currently play the main role in formation of heavy metal content in the discussed populations. Thus, lead concentrations in organs and tissues (liver, gonads, and muscle) of fish from Kuril oceanic waters was one and a half order of magnitude higher than that of pink salmon from the Sea of Japan ^33^.

If the global anthropogenic mercury pollution of terrestrial and aquatic ecosystems continues, coastal food webs in the zone of influence of the East Pacific platform will be at most risk of toxicological effects.

## Material and methods

Animal fur was used in the study based on evidence from Treu et al. ^34^ that hair samples reflect levels of total mercury (THg) of the internal tissues of predatory mammals. The material was collected in the Primorsky (n = 24) and Khabarovsk (n = 4) territories and the Amur Region (n = 1) in 2004–2014 (Fig. 1). Fur of 29 tigers was obtained from animal hides seized from poachers (according to the Rosprirodnadzor regulations) or found dead and stored under conditions that allow the use of these hides for mercury analysis ^20^. The archived samples of tiger fur were kept in isolated dense multi-layer polyethylene packages in a closed room to prevent the samples from mercury contamination. They were treated with naphtalene (pesticide) to preserve from pests.

The division of tigers, living mainly in the forests of the Sikhote-Alin mountain system (Fig. 1), into two groups associated with the eastern and western macro slopes of this system and, correspondingly, with the coast (basin of the Sea of Japan) (n = 16) and with the inland area ( Amur River basin) (n = 13) is rightful. First, “transitions of tigers from the western Sikhote-Alin macro slope to the eastern and vice versa are limited if existing at all” ^13^. Apparently, only a part of the dispersing individuals travels over the main ridge, travelling over long distances (for example, 103 km ^26^). Secondly, tigers of the eastern Sikhote-Alin macro slope are characterized by the absence ^13^ or low infection rate with oriental lung fluke (*Paragonimus westermani*) ^35^. Nevertheless, genetically, tigers of different Sikhote-Alin macro slopes represent one population, but they differ from tigers of the southwestern Primorye ^36^.

Since the molting of the tiger takes place twice a year in spring and autumn ^13,37^, we have also divided the animal samples into two groups: with autumn fur (died in November–February) (n = 18) and with predominantly spring fur (those who died in March–June) (n = 9). One fur sample belonged to a tiger with undetermined time of death. There was no information on sex of four samples, and on age for two.

In addition, we also examined the mercury content in lichens (*Usnea* sp.)—the bioindicators of atmospheric deposition, eaten by deer ^3^ (N = 56), which were collected on the coast and the inland area at a distance of about 80–100 km from the coast (Fig. 1A). Lichens were collected with clean hands, wiping them and the cutting tools with methanol between samples, and samples were stored in two polyethylene storage bags.

We measured the total concentration of Hg (THg) in fur and lichens, a convenient substitute for MMHg ^3^. The mercury presence in all samples was determined on the mercury analyzer RA-915M (Lumex) with PYRO add-on attachment. Each sample was measured twice and an average individual value was taken. To control the measurement accuracy of the device, we used Certified Reference Materials with a known concentration of mercury (CRMs): DORM—4 (Fish protein certified reference material for trace metals)—0.412 ± 0.036 mgHg/kg, DOLT—5 (Dogfish Liver Certified Reference Material for Trace Metals and other Constituents)—0.44 ± 0.18 mgHg/kg (National Research Council Canada and Institute of environmental chemistry, Ottawa, Canada). The accuracy of the device was monitored every 30 measurements (relative percent difference (RPD) < 20%). Such CRMs are used in the analysis of biological tissues, including fur, and are considered sufficient for detecting trends. The principle of operation of the RA-915M analyzer is that the matrix (structure) of the tissue does not matter to it. Since everything is burned in it totally and integrated into the final signal.

Statistical analyzes were performed using Stat Soft Statistica 12.0 and Microsoft Excel 2016 software. Arithmetic means (AM), error of mean (SE), median, standard deviations (SD), lower/upper quartile and minimum / maximum (ranges) were calculated. Distribution of empirical data on THg concentrations in the hair of the studied animals and lichens diverged from the expected normal distribution, as shown by the Kolmogorov–Smirnov test with Lilliefors correction. Therefore, in comparisons of mean values of THg concentration, nonparametric Mann–Whitney U tests were used. Statistical significance was determined at p < 0.05.

### Compliance with ethical standards

All applicable international, national, and/or institutional guidelines for the care and use of animals were followed. Live animals are not included in our study. All methods were carried out in accordance with relevant guidelines and regulations.
